# Compartment‐based reconstruction of 3D acquisition‐weighted ^31^P cardiac magnetic resonance spectroscopic imaging at 7 T: A reproducibility study

**DOI:** 10.1002/nbm.4950

**Published:** 2023-05-04

**Authors:** Andrew Tyler, Jane Ellis, Justin Y. C. Lau, Jack J. Miller, Paul A. Bottomley, Christopher T. Rodgers, Damian J. Tyler, Ladislav Valkovič

**Affiliations:** ^1^ Department of Physiology, Anatomy & Genetics University of Oxford Oxford UK; ^2^ Oxford Centre for Clinical Magnetic Resonance Research (OCMR), RDM Cardiovascular Medicine University of Oxford Oxford UK; ^3^ Department of Physics, Clarendon Laboratory University of Oxford Oxford UK; ^4^ The MR Research Centre & The PET Research Centre Aarhus University Aarhus Denmark; ^5^ The Division of MR Research Johns Hopkins Medicine Baltimore Maryland USA; ^6^ Wolfson Brain Imaging Centre University of Cambridge Cambridge UK; ^7^ Department of Imaging Methods, Institute of Measurement Science Slovak Academy of Sciences Bratislava Slovakia

**Keywords:** ^31^P, 7 T, cardiac, SLAM, SLIM, spectroscopy

## Abstract

Even at 7 T, cardiac ^31^P magnetic resonance spectroscopic imaging (MRSI) is fundamentally limited by low signal‐to‐noise ratio (SNR), leading to long scan times and poor temporal and spatial resolutions. Compartment‐based reconstruction algorithms such as magnetic resonance spectroscopy with linear algebraic modeling (SLAM) and spectral localization by imaging (SLIM) may improve SNR or reduce scan time without changes to acquisition. Here, we compare the repeatability and SNR performance of these compartment‐based methods, applied to three different acquisition schemes at 7 T. Twelve healthy volunteers were scanned twice. Each scan session consisted of a 6.5‐min 3D acquisition‐weighted (AW) cardiac ^31^P phase encode‐based MRSI acquisition and two 6.5‐min truncated k‐space acquisitions with increased averaging (4 × 4 × 4 central k‐space phase encodes and fractional SLAM [fSLAM] optimized k‐space phase encodes). Spectra were reconstructed using (i) AW Fourier reconstruction; (ii) AW SLAM; (iii) AW SLIM; (iv) 4 × 4 × 4 SLAM; (v) 4 × 4 × 4 SLIM; and (vi) fSLAM acquisition–reconstruction combinations. The phosphocreatine‐to‐adenosine triphosphate (PCr/ATP) ratio, the PCr SNR, and spatial response functions were computed, in addition to coefficients of reproducibility and variability. Using the compartment‐based reconstruction algorithms with the AW ^31^P acquisition resulted in a significant increase in SNR compared with previously published Fourier‐based MRSI reconstruction methods while maintaining the measured PCr/ATP ratio and improving interscan reproducibility. The alternative acquisition strategies with truncated k‐space performed no better than the common AW approach. Compartment‐based spectroscopy approaches provide an attractive reconstruction method for cardiac ^31^P spectroscopy at 7 T, improving reproducibility and SNR without the need for a dedicated k‐space sampling strategy.

## Graphical abstract

Compartment‐based reconstruction algorithms incorporate a segmentation‐mask (A) into the reconstruction of phase‐encoded MRSI data to generate one spectrum per compartment (B). We show that these algorithms can offer improved PCr SNR and PCr/ATP ratio reproducibility, over a single‐voxel FT approach (D), when applied to the same acquisition‐weighted ^31^P cardiac dataset.

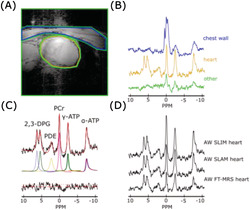

**Abbreviations**
2,3‐DPG: 2,3‐diphosphoglycerate
AMARES: advanced method for accurate, robust, and efficient spectral fitting
ATP: adenosine triphosphate
AW: acquisition‐weighted
BMI: body mass index
CoR: coefficient of reproducibility
CoV: coefficient of variation
CRLB: Cramér–Rao lower bound
DRESS: depth‐resolved surface coil spectroscopy
fSLAM: fractional SLAM
FOV: field of view
FT: Fourier transform
ISIS: image selected in vivo spectroscopy
MRS: magnetic resonance spectroscopy
MRSI: magnetic resonance spectroscopic imaging
OXSA: Oxford spectroscopy analysis
PCr: phosphocreatine
PRESS: point‐resolved spectroscopy
PSF: point spread function
SD: standard deviation
SLAM: magnetic resonance spectroscopy with linear algebraic modeling
SLIM: spectral localization by imaging
SLOOP: spatial localization with optimal point spread function
SNR: signal‐to‐noise ratio
SRF: spatial response function
STEAM: stimulated echo acquisition mode
UTE: ultrashort echo time

## 1 INTRODUCTION

Magnetic resonance spectroscopy (MRS) of high‐energy phosphorus metabolites provides a unique insight into cardiac metabolism.^1^ Of particular interest is the phosphocreatine‐to‐adenosine triphosphate metabolite ratio (PCr/ATP), which differs between healthy and diseased hearts.^2^, ^3^ Localization strategies include selective excitation of only the heart, for example, through coil design or sequences such as ISIS,^4^ PRESS,^5^ DRESS,^6^ or STEAM,^7^ or most commonly for human cardiac studies, Fourier transform‐based magnetic resonance spectroscopic imaging (FT‐MRS) reconstruction of phase‐encoded data.^8^

Unfortunately, all ^31^P MRS techniques are limited by the intrinsically low ^31^P concentrations found in vivo, which makes it difficult to achieve adequate signal‐to‐noise ratio (SNR). Localization to small volumes can degrade the achievable SNR further, because the smaller volumes contain fewer ^31^P nuclei for a given metabolite concentration. Additionally, in the case of single voxel techniques, there is the potential for a mismatch between the shape of the anatomical region of interest and the cuboidal voxel, and for Fourier transform (FT)‐based methods, SNR losses can be associated with the shape of the spatial response function (SRF). Low SNR may reduce the reproducibility of ^31^P MRS, because it negatively impacts the accuracy of fitting a model, in order to determine metabolite content, to the acquired spectra. Increasing the field strength of the magnet from 1.5 or 3 to 7 T can increase SNR and improve reproducibility^9^, ^10^; however, even at 7 T, ^31^P spectroscopy is still an inherently SNR‐limited technique.

Translation of ^31^P magnetic resonance spectroscopic imaging (MRSI) techniques, from the development stage to clinical research, faces a number of challenges. Patient cohorts may produce significantly different results to healthy volunteers, because of physiological differences (age, heart rate, body mass index [BMI], etc.), making validation in patients essential; however, long scan times make it difficult to add experimental acquisitions to already long clinical research protocols. Therefore, improved reconstruction techniques, which could be applied to ^31^P acquisitions already validated for use in clinical research, would be of great value as a means to improve SNR and repeatability without changing the clinical scan protocol, allowing the current reconstruction algorithm to serve as a fail‐safe.

FT‐based MRSI reconstruction^11^ of acquisition‐weighted (AW) data is commonly used at the higher field strengths of 3 and 7 T^8^, ^10^, ^12^, ^13^, ^14^ for ^31^P MRSI. However, alternative compartment‐based reconstruction strategies^15^, ^16^, ^17^, ^18^, ^19^, ^20^, ^21^ could potentially improve spectral SNR and PCr/ATP repeatability, without needing to change this already validated AW ^31^P scan. These strategies, which separate out the ^31^P signal into arbitrarily shaped user‐defined compartments based on relatively homogeneous anatomical structures (e.g., heart,^22^ liver,^23^ skeletal muscle,^24^ etc.), have been under development for several years. However, these techniques may be adversely affected by the increased inhomogeneities in the static (B_0_) and RF (B_1_) fields found at 7 T, compared with 1.5 or 3 T.

The magnetic resonance spectroscopy with linear algebraic modeling (SLAM) and spatial localization with optimal point spread function (SLOOP) compartment‐based techniques have been validated for cardiac ^31^P MRSI at the lower field strengths of 1.5^25^ and 3 T^26^, ^27^; however, method‐specific sequences were used. In this work, we compare the performance at 7 T of the compartment‐based reconstruction algorithms used in these methods, the SLAM and spectral localization by imaging (SLIM) algorithms, respectively, against FT‐based ^31^P MRSI reconstruction when applied to AW, uniform, and per‐volunteer optimized phase encode datasets.

## 2 COMPARTMENT‐BASED RECONSTRUCTION ALGORITHMS

The SLAM and SLIM algorithms formulate the problem of reconstructing spectra from C compartments as the solution to a system of M≥C equations that can be developed from the FT MRI signal equation. For one (spatial) dimension, the magnetic resonance spectroscopic imaging (MRSI) localization problem can be written as a continuous FT (CFT)^20^:

(1) $$s\left(k,t\right)=\iint \rho \left(\;x,f\right)e^{-i2\pi \left.\left(\mathrm{kx}+\text{ft}\right.\right)}\;df\;dx,$$

where $s\;\left(k,t\right)$ is the received signal at k‐space position k and time t and $\rho \left(x,f\right)$ is the spectral density at spatial position x (normalized by 1/field of view [FOV]) and frequency f. In the SLAM reconstruction, this equation is discretized to give a discrete FT, and a time‐domain FT applied to give

(2) $$s\left(k\right)=\sum_{m=1}^{M}\rho \left(x_{m}\right)e^{-i2\mathrm{\pi k}x_{m}}.$$

For simplicity, $\text{FT}\left[s\left(k,t\right)\right]$ is denoted $s\left(k\right)$ and $\rho \;\left(x,f\right)$ as $\rho \left(x\right)$, noting that $s\left(k\right)$ and $\rho \left(x\right)$ are length N row vectors. This allows the equation to be expanded as

(3) $${\left[\begin{array}{c} s\left(k_{1}\right)\\ s\left(k_{2}\right)\\ \vdots \\ s\left(k_{M}\right) \end{array}\right]}_{M\times N}={\left[\begin{array}{cccc} R_{1}e^{-i2\pi k_{1}x_{1}} & R_{1}e^{-i2\pi k_{1}x_{2}} & \cdots & R_{1}e^{-i2\pi k_{1}x_{M}}\\ R_{2}e^{-i2\pi k_{2}x_{1}} & R_{2}e^{-i2\pi k_{2}x_{2}} & \cdots & R_{2}e^{-i2\pi k_{2}x_{M}}\\ \vdots & \vdots & \ddots & \vdots \\ R_{M}e^{-i2\pi k_{M}x_{1}} & R_{M}e^{-i2\pi k_{M}x_{2}} & \cdots & R_{M}e^{-i2\pi k_{M}x_{M}} \end{array}\right]}_{M\times M}\times {\left[\begin{array}{c} \rho \left(x_{1}\right)\\ \rho \left(x_{2}\right)\\ \vdots \\ \rho \left(x_{M}\right) \end{array}\right]}_{M\times N},$$

where M is the number of phase encodes and N the number of points recorded in the readout. As a further refinement to the SLAM technique, enabling the use of AW datasets, we introduce $R_{m}$, the number of repeats of the mth phase encode here. Each row in the second matrix is weighted by $R_{m}$ to account for the effect on the signal magnitude of adding up the varying number of repeats for the different phase encodes. For conventional SLAM implementations, $R_{m}=1$.

The matrix equation can be written in short hand as $S_{M\times N}={\mathrm{PE}}_{M\times M}\times \rho_{M\times N}$, where S is the acquired data, PE is the phase encode operator defined by the second matrix in Equation (3), and ρ is the unencoded spectra for each spatial location $x_{m}$.

The SLAM method reconstructs spectra from C≤M user‐defined compartments by summing (with a normalization factor) the columns of the PE matrix, which correspond to x positions within each of the compartments, thereby reducing the dimensions of the reconstruction to

(4) $$S_{M\times N}={\mathrm{PE}}_{M\times C}\times \rho_{C\times N}.$$

Now that M≥C, and therefore ${\mathrm{PE}}_{M\times C}$ is overdetermined, a reduced gradient set of $M^{\prime }\geq C$ phase encodes may be used. To maximize SNR, the phase encodes are chosen from central k‐space, and Equation (4) reduces to

(5) $$S_{M^{\prime }\times N}={\mathrm{PE}}_{M^{\prime }\times C}\times \rho_{C\times N}.$$

This equation is solved by premultiplying both sides by the Moore–Penrose pseudoinverse of ${\mathrm{PE}}_{M\prime \times C}$. Because the number of phase encodes acquired ( $M^{\prime }$) can be set to any value greater than or equal to C, acceleration factors of up to M/C compared with a conventional M gradient step MRSI experiment can be achieved.

The SLIM reconstruction algorithm separates the MRSI equation (Equation 1) into C integrals, corresponding to C compartments, giving

(6) $$s\left(k,t\right)=\int_{\text{comp}\;1}\xi \left(x,t\right)e^{-i2\mathrm{\pi kx}}dx+\ldots +\int_{\text{comp}\;C}\xi \left(x,t\right)e^{-i2\mathrm{\pi kx}}\;dx,$$

where $\xi \left(x,t\right)$ is defined as the time‐domain FT of $\rho \left(x,f\right)$.

(7) $$\xi \left(x,t\right)=\int_{-\infty }^{\infty }\rho \left(x,f\right)e^{-i2\mathrm{\pi ft}}df.$$

If $\xi \left(x,t\right)$ depends only on t within each compartment (i.e., the compartment is homogeneous), we can substitute in the compartment signal $\xi_{c}\left(t\right)$ for $\xi \left(x,t\right)$, giving

(8) $$s\left(k_{m},t\right)=\sum_{c=1}^{C}g_{\mathrm{mc}}\xi_{c}\left(t\right),\;m=1,\ldots ,M^{\prime },$$

where $M^{\prime }$ is the number of phase encode steps recorded and $g_{\mathrm{mc}}$ is defined in 1D as^15^

(9) $$g_{\mathrm{mc}}=R_{m}\int_{\text{comp}\;c}e^{-i2\pi k_{m}x}\;dx.$$

Here, $R_{m}$ is the number of repeats of the $m^{\mathrm{th}}$ phase‐encoding step in order to account for acquisition weighting, $k_{m}$ is the k‐space position of the $m^{\mathrm{th}}$ phase‐encoding step, and comp c is the spatial extent of the c^th^ compartment. Because Equation (6) is at no point discretized, the CFT forms the basis of the SLIM equation, with the step between Equations (6) and (8) introducing a homogeneity condition for each of the compartment spectra.

This gives the matrix equation

(10) $$S_{M^{\prime }}\left(t\right)=G\xi_{C}\left(t\right).$$

When solved by premultiplying both sides by H, where HG=I, this gives the separated signal for each compartment, which can be Fourier transformed to give each compartment's spectra ( $\rho_{c}$). Because this equation is overdetermined for M>C, the number of phase encodes ( M) may be reduced to $M^{\prime }\geq C$ as in SLAM.

The fractional SLAM (fSLAM) method improves the localization properties of SLAM by numerically optimizing the fractional k‐space coordinates of the phase encodes^16^, ^20^ based on the compartmentalization mask. Because a localizer must be acquired to segment the compartments, this optimization must be done scanner‐side at the start of the examination, increasing the time in the scanner.

An essential metric for understanding the SLAM and SLIM algorithms is the SRF, which gives the contribution of the metabolites at each point in the FOV to each compartment's spectra and allows the effect on the reconstruction of changing the acquired phase encodes to be quantified. SRFs can be calculated for both the SLAM and SLIM reconstructions, by performing the reconstruction for each point in space, with the following formula:

(11) $$\beta_{c}\left(x\right)=\sum_{m=1}^{M^{\prime }}h_{\mathrm{cm}}R_{m}e^{-i2\pi k_{m}x}$$

Here, $\beta_{c}\left(x\right)$ is the $c^{\mathrm{th}}$ compartment's SRF (with magnitude and phase components), $h_{\mathrm{cm}}$ is the ${\mathrm{cm}}^{\mathrm{th}}$ element of H, and H is the pseudoinverse of PE or G for SLAM and SLIM, respectively. $R_{m}$ is the number of repeats of the $m^{th}$ phase encode, to allow for the effect of acquisition weighting to be calculated; for conventional SLAM and SLIM implementations, $R_{m}=1$.

In a “perfect” theoretical experiment (with completely uniform B_0_, B_1_, and metabolite distribution within each compartment), the sum of a compartment's SRF over each of the other compartments multiplied by the signal distribution is exactly zero, providing perfect localization. In a real setting, however, the reconstructed spectra of a compartment, subject to B_0_, B_1_, and compartment inhomogeneity, can be calculated as

(12) $$\rho {֮}_{c}\propto \int_{\mathrm{FOV}}\beta_{c}\left(x\right)∆B_{1}\left(x\right)\phi \left(x\right)\rho \left(x,f-∆f\left(x\right)\right)\;dx,$$

where $\rho {֮}_{c}$ is the reconstructed compartment spectrum for compartment c, $∆B_{1}\left(x\right)$ the relative B_1_ distribution, $\phi \left(x\right)$ the phase distribution, caused by B_0_ inhomogeneity, and $\rho \left(x,f-∆f\left(x\right)\right)$ the true, spatially inhomogeneous, spectra of the spins at spatial position x, with an added spatial component $∆f\left(x\right)$ to reflect B_0_ inhomogeneity.

An SRF that assumes the “worst case” for spectral contamination from other compartments, caused by B0 inhomogeneity (when $\arg\left[\beta_{c}\left(x\right)\right]=\pm \phi \left(x\right)$), can be calculated by taking the magnitude of the SRF, which is used in the cost functions of SLOOP^16^ and fSLAM^20^ when optimizing the phase‐encoding scheme. If the B_1_ distribution is known, this magnitude SRF can then be weighted by B_1_ to provide a guide to the likely signal contamination caused by field inhomogeneity.

The effect of inhomogeneity on the signal from within a compartment on that compartment's spectra is more difficult to quantify, with the major source of concern B_0_ inhomogeneity. In the absence of B_0_ inhomogeneity, the contribution of signal from within a compartment to the compartment's spectra will be a weighted sum of that signal. In the presence of severe B_0_ inhomogeneity, however, there can also be signal cancellation, which could, if the metabolite distribution within the compartment is also highly heterogeneous, result in incorrect metabolite distributions, in addition to severely degraded SNR and poor spectral linewidths.

Numerous strategies to minimize the impact of inhomogeneity exist, with the optimal strategy depending largely on application and available SNR. These include the incorporation of a B_0_ map for BSLIM,^18^ per‐volunteer phase encode optimization for fSLAM^20^ and SLOOP,^16^ compartment subdivision during reconstruction,^19^ and higher resolution acquisitions.

## 3 METHODS

**FIGURE 1:**
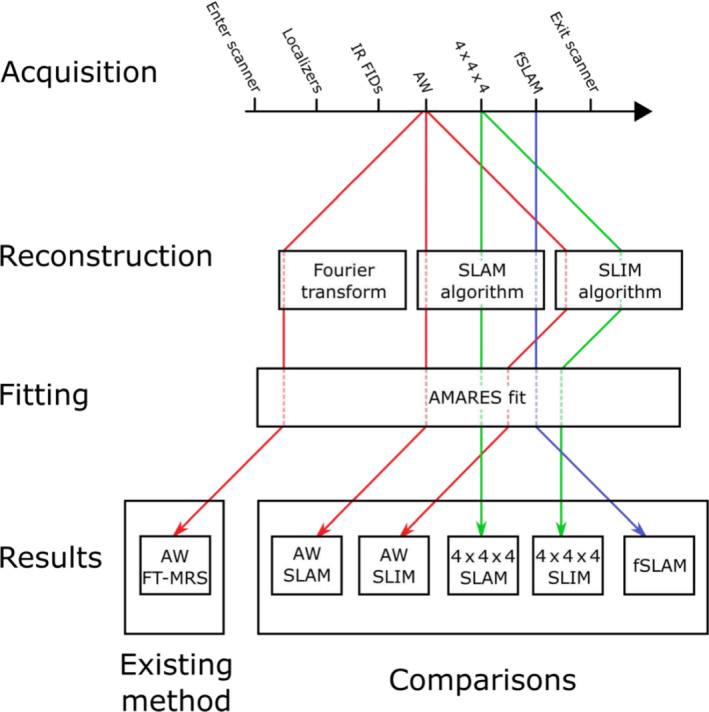
An overview of the data pathways for each acquisition/reconstruction combination considered from acquisition through reconstruction and fitting to results. AMARES, advanced method for accurate, robust, and efficient spectral fitting; AW, acquisition‐weighted; fSLAM, fractional SLAM; FT‐MRS, Fourier transform‐based magnetic resonance spectroscopic imaging; IR FIDs, inversion recovery free induction decay acquisitions; SLAM, magnetic resonance spectroscopy with linear algebraic modeling; SLIM, spectral localization by imaging.

Twelve healthy volunteers (six females/six males, age 29 ± 4 years, BMI 23 ± 3 kg/m^2^) were recruited and gave informed consent for this study approved by our research ethics committee. The reproducibility of each method was quantified using a test–retest procedure, where each volunteer was scanned twice (each scan consisted of three ^31^P MRSI acquisitions + ^1^H anatomical localizers), with the volunteer taking a short break outside of the scan room between scans. Each method consisted of a combination of a phase‐encoding scheme and reconstruction algorithm. The phase‐encoding schemes were: 3D AW k‐space points (AW), 4 × 4 × 4 central integer k‐space points (4 × 4 × 4) and 4 × 4 × 4 k‐space points whose positions were optimized with the fSLAM algorithm^20^ (fSLAM). The reconstruction algorithms were the SLAM, SLIM, and FT‐MRS algorithms. This gave a total of six plausible combinations: AW FT‐MRS, AW SLAM, AW SLIM, 4 × 4 × 4 SLAM, 4 × 4 × 4 SLIM, and fSLAM SLAM (fSLAM SLAM is henceforth referred to as fSLAM). The acquisition–reconstruction combinations are detailed in Table 1 and displayed graphically in Figure 1.

**TABLE 1:** Summary of acquisitions and reconstructions performed.

Technique name	Acquisition	Reconstruction algorithm	Phase encodes	Averages	Time (min)
AW FT‐MRS	AW k‐space	Fourier transform	8 × 16 × 8	4	6.5
AW SLAM		SLAM			
AW SLIM		SLIM			
4 × 4 × 4 SLAM	Central integer k‐space	SLAM	4 × 4 × 4	6	6.5
4 × 4 × 4 SLIM		SLIM			
fSLAM	fSLAM optimized k‐space	SLAM	4 × 4 × 4	6	6.5

Abbreviations: AW, acquisition‐weighted; fSLAM, fractional SLAM; FT‐MRS, Fourier transform‐based magnetic resonance spectroscopic imaging; SLAM, magnetic resonance spectroscopy with linear algebraic modeling; SLIM, spectral localization by imaging.

### 3.1 SRF simulations

**FIGURE 2:**
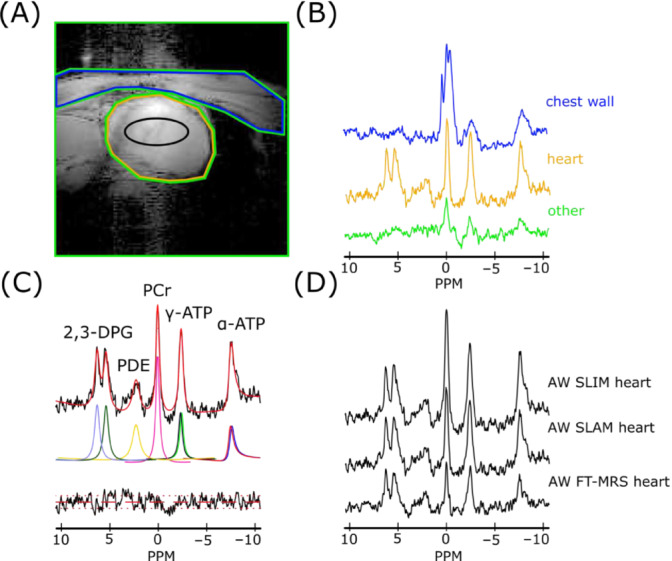
(A) A sample segmentation map of one slice of the heart, showing (blue) chest wall, (orange) heart and (green) other. The black ring represents the midseptal voxel (64% threshold of voxel PSF) of the AW FT‐MRS method (a full segmentation is shown in the supporting information). (B) Sample spectra for each of the compartments in (A) reconstructed using the SLAM algorithm and AW phase‐encoding scheme (AW SLAM). (C) Fit, using the OXSA toolbox of the heart compartment spectra in (B). (D) AW SLIM and AW SLAM spectra for the heart compartment in (A) with the AW FT‐MRS spectra of the midseptal voxel. 2,3‐DPG resonance intensity is proportional to the quantity of blood in the sensitive volume and used for the blood correction. Spectra in (B) and (D) are normalized by noise. 2,3‐DPG, 2,3‐diphosphoglycerate; ATP, adenosine triphosphate; AW, acquisition‐weighted; FT‐MRS, Fourier transform‐based magnetic resonance spectroscopic imaging; OXSA, Oxford spectroscopy analysis; PCr, phosphocreatine; PDE, phosphodiesters; PPM, parts per million; PSF, point spread function; SLAM, magnetic resonance spectroscopy with linear algebraic modeling; SLIM, spectral localization by imaging.

SRFs for a representative compartmentalization mask (Figure 2A) were calculated for each method. The SRFs were calculated using the formulas described by Hu et al.^15^ and Zhang et al.^20^ and weighted with receive coil sensitivity calculated using the Biot–Savart law.

### 3.2 Data acquisition

All acquisitions were undertaken on a Siemens MAGNETOM 7‐T whole‐body MRI scanner (Siemens Healthineers, Erlangen, Germany) with volunteers in the supine position, as previously described.^9^ The RF coil used was a dual‐tuned (^31^P/^1^H) transmit–receive quadrature, surface coil consisting of a ^31^P coil (two 15‐cm loops, with overlap decoupling), and a single ^1^H loop (10 cm in diameter).^28^ Two‐chamber, four‐chamber, short‐axis, and FT‐MRS–matched ^1^H images were acquired with a fast low‐angle shot (FLASH) sequence.^29^ The coil position was determined from images showing the location of four cod‐liver oil capsules and one central phenylphosphonic acid fiducial embedded in the coil housing during imaging, and the transmit flip angle was calculated from a series of inversion recovery free induction decay acquisitions of the central fiducial, as previously described.^9^

Three different ^31^P acquisitions, corresponding to the AW, 4 × 4 × 4, and fSLAM phase‐encoding schemes, were applied for the comparative studies. The ^31^P acquisitions consisted of a 3D ultrashort echo time (UTE)‐FT‐MRSI sequence,^30^ where the excitation was achieved with a shaped RF pulse, numerically optimized for excitation homogeneity^8^; the excitation bandwidth was approximately 2 kHz. The excitation was placed with its center frequency at +266 Hz (towards the 2,3‐diphosphoglycerate [2,3‐DPG] resonance) relative to the PCr resonance. The repetition time (TR) was 1 s.

**FIGURE 3:**
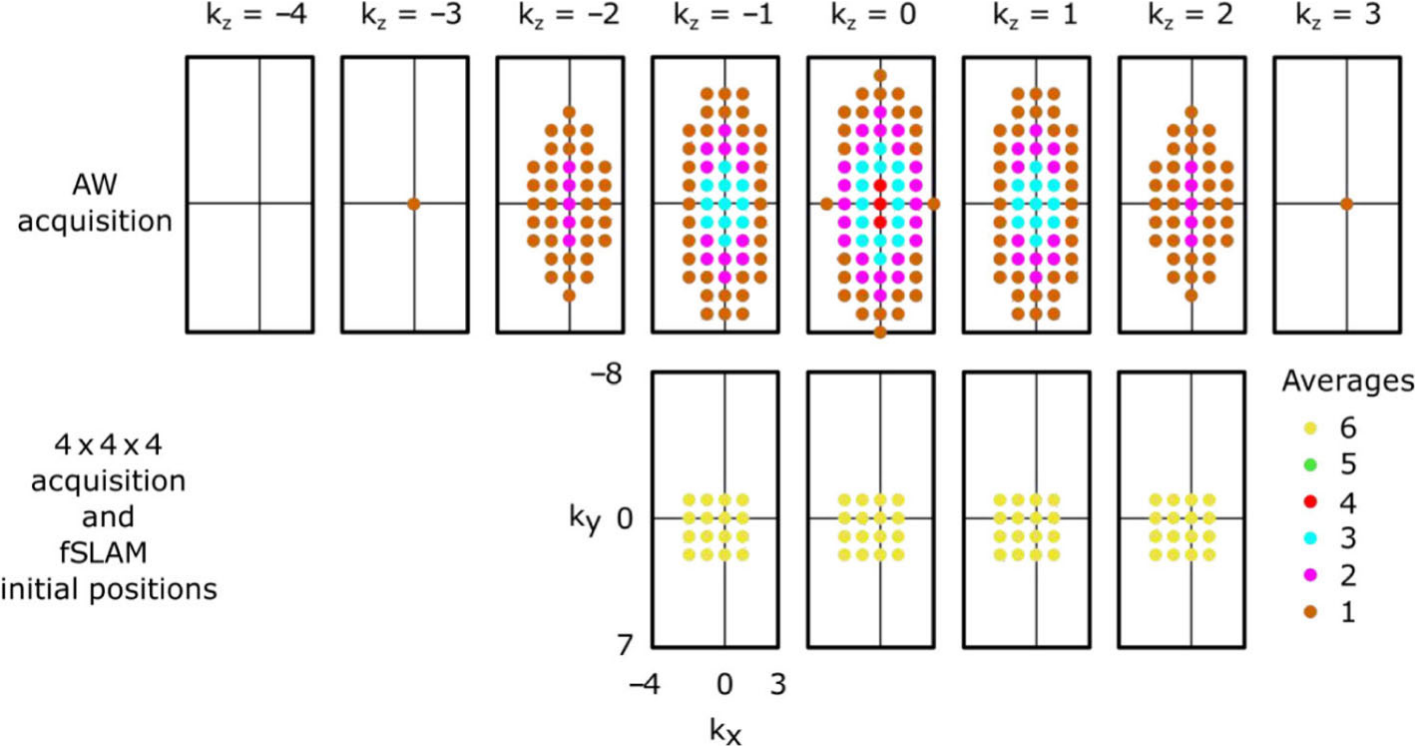
k‐space coordinates of the phase encodes used in the three ^31^P acquisitions; all k‐space coordinates are normalized by the FOV to give integer values; the fSLAM initial positions refer to the k‐space values used as the starting conditions for the fSLAM optimization algorithm. The final fractional fSLAM optimized k‐space coordinates are different for each compartmentalization mask and are defined here by a mesh of k_x_, k_y_, and k_z_ values. For the compartmentalization mask used in Figure 2, these are: k_x_ = −1.94, −0.99, 0.01, 1.08; k_y_ = −2.19, −1.11, −0.01, 1.13; k_z_ = −1.83, −0.96, 0.00, 0.93. FOV, field of view; fSLAM, fractional SLAM; SLAM, magnetic resonance spectroscopy with linear algebraic modeling.

The phase‐encoding schemes used in the acquisitions are summarized in Table 1. All had 3D phase encoding, a FOV of 240 × 240 × 200 mm, and took 6.5 min to complete. The acquisitions were in the short‐axis orientation, covering the heart, with identical FOV placement for all acquisitions in each volunteer. The first phase‐encoding scheme (AW) was 3D AW, with a matrix size of 8 × 16 × 8 (x,y,z) and four averages at k = (0, 0, 0) (396 total readouts), to give a nominal voxel size of 30 × 15 × 25 mm^3^ with FT reconstruction. The second scheme (4 × 4 × 4) acquired data at phase encodes corresponding to the central 4 × 4 × 4 k‐space coordinates of the AW scheme, but with six averages at all k‐space points (384 total readouts). The final phase‐encoding scheme (fSLAM) consisted of 4 × 4 × 4 phase encodes with six averages at each k‐space point; however, the exact fractional k‐space values were optimized using the fSLAM algorithm for intercompartmental and intracompartmental leakage according to Zhang et al.^20^ The acquisition order was fixed to allow the fSLAM coordinates to be calculated while the AW and 4 × 4 × 4 acquisitions ran and for scan planning to be accomplished using the AW sequence image overlay. The k‐space coordinates used for each acquisition are detailed in Figure 3.

To enable calculation of the fSLAM coordinates while the AW and 4 × 4 × 4 phase‐encoding scheme acquisitions completed, the degrees of freedom in the optimization were reduced to 12, after forming a 1D vector composed of the k‐space coordinates in each orthogonal direction. The optimizations were performed with the Matlab programming language (MathWorks, Natick, MA, USA) and took approximately 2.5 min to run (2 × Intel Xeon Gold 6252, 48 cores/96 threads; 768 GiB RAM).

### 3.3 Data Analysis

Reconstruction of the spectra was performed off‐line using raw data (exported with the Siemens TWIX utility) for all methods. The SLIM and SLAM algorithms were implemented in the Matlab programming language according to the equations presented by Hu et al.^15^ and Zhang et al.,^20^ respectively, in addition to a standard FT‐MRS reconstruction. The SLAM and SLIM reconstructions included an additional weighting factor ( $R_{m}$) to allow the reconstruction of data with a nonuniform number of averages at each k‐space point. An alternative method for solving the SLAM equation (SLAM*)^26^ did not produce a significant improvement here, and was not used. For cardiac FT‐MRS reconstructions, a single midseptal voxel spectrum was chosen,^8^, ^10^ because summing multiple voxels produced contamination artefacts (see the supporting information). The compartmentalization masks used in the reconstruction were defined during acquisition and consisted of three compartments: heart, chest wall, and other (see Figure 2A). Blood was not segmented because of the impact of cardiac and respiratory motion. If present, the heart compartment was removed from the final two slices (base of heart) to avoid aliasing of the heart compartment's SRF into the first slice. The raw reconstructed spectra from each coil element were combined with a noise‐whitened singular value decomposition coil combination,^31^ phased (zeroth and first order), apodized, and fitted with the Oxford spectroscopy analysis (OXSA) implementation of AMARES,^23^, ^32^ with an identical protocol used for all spectra. PCr/(γ‐)ATP ratios were blood‐ and saturation‐corrected, as previously reported.^9^ The SNR of the PCr resonance and the Cramér–Rao lower bound (CRLB) of the PCr fit were calculated. The coefficient of reproducibility (CoR) was defined as^10^

(13) $$\mathrm{CoR}={\mathrm{SD}}_{\text{intrasubject}}\times 1.96,$$

where ${\mathrm{SD}}_{\text{intrasubject}}$ is the standard deviation ( SD) of the signed difference in the PCr/ATP ratio between scans of the same subject with the same technique. The coefficient of variation (CoV) was defined as the SD of the PCr/ATP ratio across all datapoints for a technique (both repeats) divided by the mean value of the PCr/ATP ratio for that technique. Spectra from one participant with excessive movement (seen by the operator during the scan), and three of the remaining individual spectra, which showed very high liver contamination leading to the blood correction failing, were excluded from the analysis (the corresponding spectra from the other repeat were also excluded from paired analysis by necessity). Errors are reported as ±1 SD. All comparisons were with the AW FT‐MRS method, which is considered the standard method at our center. Pairwise two‐tailed Wilcoxon signed‐rank tests with 1% significance level (5% plus Bonferroni correction) were used for all comparisons.

## 4 RESULTS

An overview of the results at each stage of the reconstruction process is given in Figure 2. It shows a representative segmented slice through the heart (a full segmentation stack is shown in the supporting information), the spectra generated by reconstructing the AW dataset corresponding to that volunteer using the SLAM algorithm, a sample fit, and comparison of cardiac spectra reconstructed from the same AW dataset using the SLAM, SLIM, and FT‐MRS reconstruction algorithms. Clear spectral differences between the chest wall and heart compartments can be seen, in addition to an increase in SNR from the SLAM/SLIM algorithm compared with the FT‐MRS algorithm.

### 4.1 SRF simulations

**FIGURE 4:**
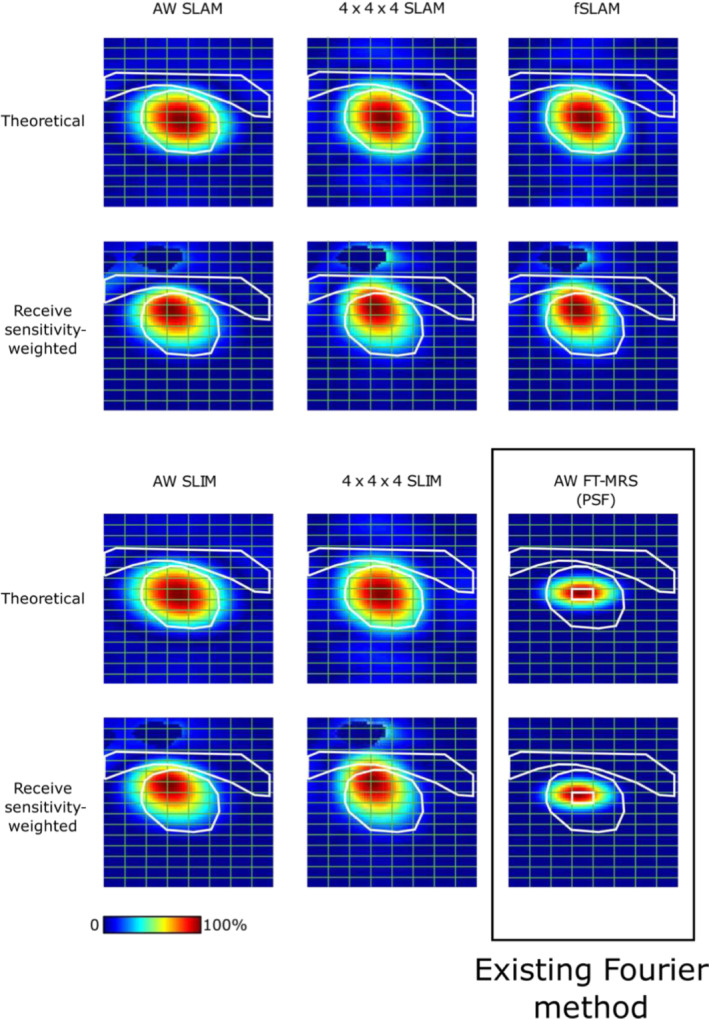
Simulated cardiac compartment SRF magnitude images for each SLAM/SLIM algorithm‐based method for a representative compartmentalization mask (Figure 2A) and AW FT‐MRS midseptal voxel PSF (same as Figure 2). Both theoretical and B_1_ receive sensitivity‐weighted SRFs are shown. The MRSI grid is shown in green, and the midseptal voxel highlighted in white in the AW FT‐MRS PSFs. The SRFs are displayed as a percentage of the maximum intensity. The coil sensitivity map applied to the SRFs was zeroed, close to the coil elements and within the coil housing, where the Biot–Savart simulation was not valid. AW, acquisition‐weighted; fSLAM, fractional SLAM; FT‐MRS, Fourier transform‐based magnetic resonance spectroscopic imaging; PSF, point spread function; SLAM, magnetic resonance spectroscopy with linear algebraic modeling; SLIM, spectral localization by imaging; SRF, spatial response function.

Representative cardiac compartment SRFs for each of the compartment‐based acquisitions are shown in Figure 4 with, and without, coil sensitivity weighting (an example B^−^_1_ receive field map is shown in the supporting information). The segmentation of the heart as a spheroid resulted in the maximum of the SRF placed on the midseptal voxel, which has previously been shown to be optimal for the AW FT‐MRS method,^10^ and extending out to the perimeter of the heart. The AW acquisitions feature an elliptical SRF, with the semi‐minor axis in the vertical y‐direction, reflecting the higher number of phase encodes in the y‐direction. The inclusion of coil sensitivity weighting to the theoretical (un‐weighted) SRFs skews the AW SLAM and AW SLIM SRFs towards the anterior wall of the heart, in the direction of the coil. The 4 × 4 × 4 SLAM and 4 × 4 × 4 SLIM SRFs both show slightly greater overspill of the cardiac compartment's SRF into the chest wall than AW SLAM and AW SLIM and a more spherical SRF. The differences between 4 × 4 × 4 SLAM and fSLAM are subtle; however, fSLAM does appear to reduce some spillover of the SRF into the chest wall, particularly once coil sensitivity is taken into account.

### 4.2 AW k‐space acquisitions

**FIGURE 5:**
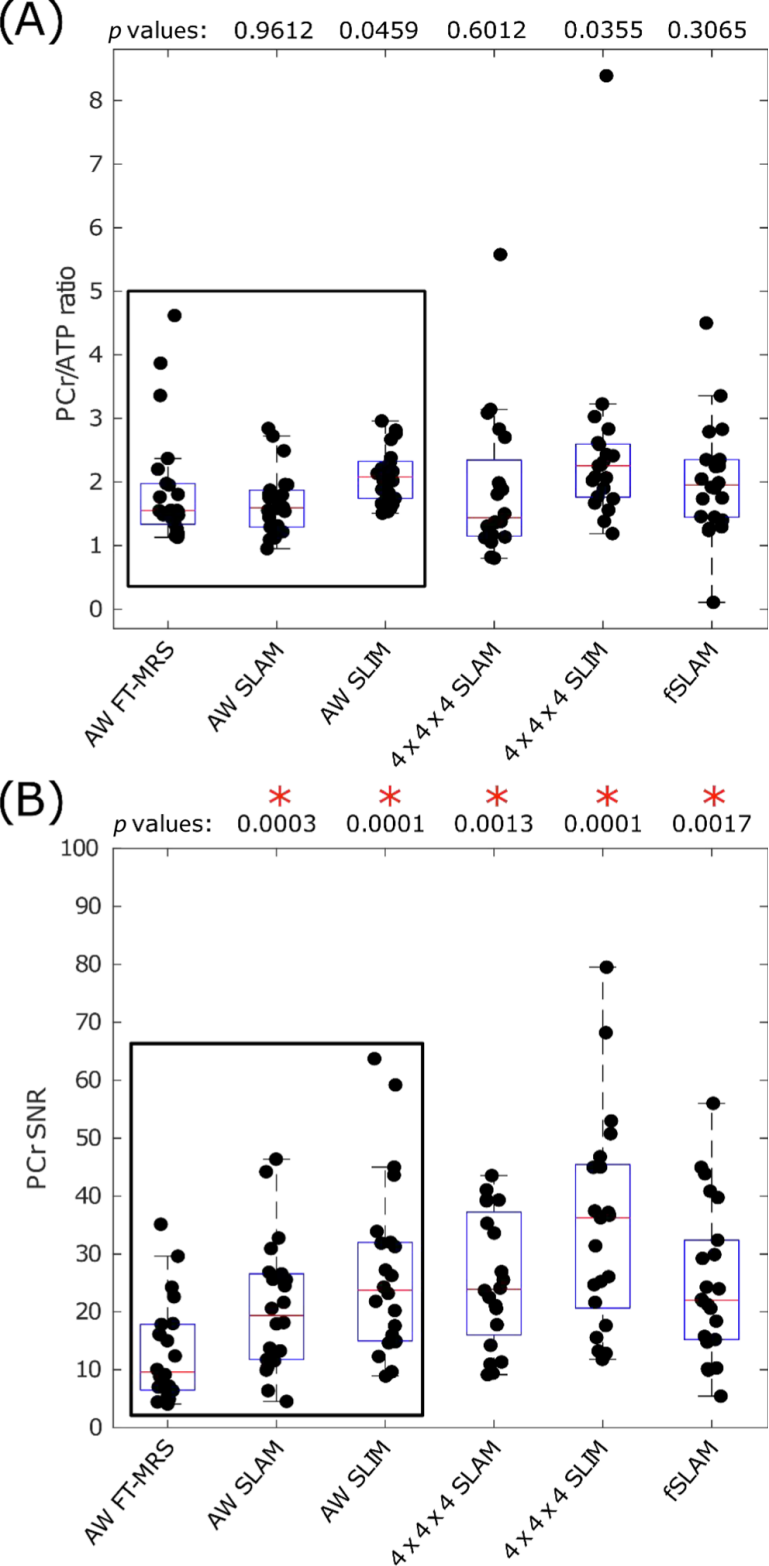
Boxplot showing (A) PCr/ATP values and (B) PCr SNR for each acquisition, median and IQR indicated by box. * indicates significant difference to the AW FT‐MRS method (Wilcoxon signed‐rank paired, α = 0.05, *p* values above boxplot). Reconstructions that use the AW phase‐encoding scheme are highlighted by the surrounding black box. PCr/ATP values are in the expected range.^8^, ^9^, ^10^, ^33^ ATP, adenosine triphosphate; AW, acquisition‐weighted; fSLAM, fractional SLAM; FT‐MRS, Fourier transform‐based magnetic resonance spectroscopic imaging; PCr, phosphocreatine; SLAM, magnetic resonance spectroscopy with linear algebraic modelling; SLIM, spectral localization by imaging; SNR, signal‐to‐noise ratio.

Figure 5 shows boxplots of the PCr/ATP ratio and PCr SNR recorded in the study. Numerical values for PCr/ATP ratio, PCr SNR, PCr linewidth, PCr fit CRLB, CoR and CoV are given in Table 2. The AW FT‐MRS method measured a PCr/ATP ratio of 1.90 ± 0.92 with a PCr SNR of 13.10 ± 8.63 and PCr linewidth of 41.8 ± 22.4 Hz. The CoR was 1.79 and CoV 0.48. By comparison, the AW SLAM and AW SLIM both had a significantly higher PCr SNR than the AW FT‐MRS method (Wilcoxon single‐pair paired, α = 0.01). Both had lower CoR and CoV than the AW FT‐MRS method. Neither AW SLAM nor AW SLIM had a significantly different PCr/ATP ratio, or PCr linewidth, compared with the AW FT‐MRS method.

**TABLE 2:** Mean values of the experimental results derived for each technique; errors are given as ±1 SD. All PCr SNR values were significantly different to the AW FT‐MRS method.

Technique	PCr/ATP ratio	PCr SNR	PCr linewidth (Hz)	PCr CRLB	CoR	CoV
AW FT‐MRS	1.90 ± 0.92	13.10 ± 8.63	41.8 ± 22.4	13.36 ± 7.37	1.79	0.48
AW SLAM	1.68 ± 0.50	20.75 ± 11.18	38.0 ± 21.5	8.08 ± 4.19	1.22	0.30
AW SLIM	2.09 ± 0.43	26.93 ± 14.95	44.9 ± 20.3	5.71 ± 2.57	1.05	0.21
4 × 4 × 4 SLAM	1.90 ± 1.13	25.43 ± 11.44	37.1 ± 18.6	7.60 ± 4.20	3.29	0.59
4 × 4 × 4 SLIM	2.46 ± 1.46	35.05 ± 18.26	58.7 ± 39.4	5.57 ± 3.26	3.49	0.59
fSLAM	2.02 ± 0.88	25.05 ± 13.29	45.9 ± 21.8	9.54 ± 10.75	2.43	0.43

Abbreviations: ATP, adenosine triphosphate; AW, acquisition‐weighted; CoR, coefficient of reproducibility; CoV, coefficient of variation; CRLB, Cramér–Rao lower bound; fSLAM, fractional SLAM; FT‐MRS, Fourier transform‐based magnetic resonance spectroscopic imaging; PCr, phosphocreatine; SLAM, magnetic resonance spectroscopy with linear algebraic modeling; SLIM, spectral localization by imaging; SNR, signal‐to‐noise ratio.

### 4.3 Truncated k‐space acquisitions

The 4 × 4 × 4 SLAM and 4 × 4 × 4 SLIM reconstructions both had a significantly higher PCr SNR than the standard AW FT‐MRS method; however, the CoV and CoR for the 4 × 4 × 4 SLAM and 4 × 4 × 4 SLIM methods were higher (worse) than for the AW FT‐MRS method. The 4 × 4 × 4 SLAM and 4 × 4 × 4 SLIM PCr/ATP ratios, and PCr linewidths, were not significantly different from the AW FT‐MRS method PCr/ATP ratio. The fSLAM method had a significantly higher PCr SNR than the AW FT‐MRS method, and a lower (better) CoV, although CoR was higher. The fSLAM method's PCr/ATP ratio and PCr linewidth were also not significantly different to the PCr/ATP ratio and PCr linewidth of the AW FT‐MRS method.

## 5 DISCUSSION

In this work, we investigated the use of SLAM and SLIM reconstruction algorithms to reconstruct a standard AW phase encode acquisition (AW SLAM and AW SLIM) at 7 T and compared this with a typical Fourier‐based reconstruction of the same AW data (AW FT‐MRS). We also evaluated two alternative acquisition phase encode schemes (with equal acquisition time) for reconstruction with the SLAM and SLIM algorithms: 4 × 4 × 4 k‐space phase encodes and fSLAM optimized k‐space phase encodes (reconstructed with the SLAM algorithm only). These additional techniques were compared with AW FT‐MRS only, as there was insufficient statistical power for a full comparison between all methods with a reasonable sample size. We found that all acquisition–reconstruction combinations delivered a significant improvement in SNR over AW FT‐MRS and that AW SLAM also improved reproducibility while maintaining the PCr/ATP ratio. However, the increase in SNR, and consequently the lower CRLBs, did not uniformly translate into reduced variance across the three acquisition datasets, suggesting that the spread in PCr/ATP ratio is dominated by factors other than SNR, such as the impact of the shape of the SRF on sensitivity to motion artefacts and compartment heterogeneity, which is an area of future study, as well as the natural biological variation across a cohort. This finding mirrors the work of Ellis et al.,^10^ where multiple septal voxels, reconstructed using the AW FT‐MRS method, were summed to improve SNR, which did not translate into improved reproducibility.

### 5.1 AW k‐space acquisitions

We recorded a PCr/ATP of 1.90 ± 0.92 using the AW FT‐MRS method, which is in good agreement with the previous literature values at 1.5, 3, and 7 T.^8^, ^9^, ^10^, ^33^ The AW FT‐MRS method had a CoR of 1.79 and a CoV of 0.48. The CoR reported by Ellis et al. (0.67),^10^ who used the same AW FT‐MRS method, is lower than our result; however, the CoV (0.38) is comparable. This is probably because of the different receive coil designs used in the two studies, where Ellis et al. used a 16‐channel receive array, which would be expected to deliver much better sensitivity and homogeneity than the two‐channel quadrature coil used in the current study.

Both of the AW SLAM and AW SLIM methods improve SNR over the AW FT‐MRS method while improving reproducibility. The PCr/ATP ratio is also maintained for both methods with AW SLAM, achieving a ratio of 1.68 ± 0.50, close to the 1.71 ± 0.65 midseptal voxel value reported by Ellis et al., and AW SLIM returning 2.09 ± 0.43, which is within the range of literature values,^8^, ^9^, ^10^, ^33^ suggesting good rejection of out‐of‐compartment signals. This is supported by the calculated SRFs, where the low magnitude of the AW SRFs inside of the chest wall suggests that inhomogeneity within the chest wall is unlikely to cause undue bias.

Because the same AW dataset is input into SLAM, SLIM and FT‐MRS reconstruction algorithms, it is likely that the SNR increase seen here is caused by the increase in coil sensitivity‐weighted tissue volume (see Figure 4) of the cardiac compartment over the single voxel used in the FT‐MRS algorithm, particularly because the SLAM reconstruction simplifies to the FT‐MRS if the compartment masks are set to the voxels of FT‐MRS. The observed SNR increase over FT‐MRS offers further reassurance to the application of SLAM and SLIM at 7 T, because if B_0_ inhomogeneity within the heart was a serious concern, SNR would be impacted through signal cancellation. Furthermore, poor B_0_ inhomogeneity within a compartment would be expected to cause poor spectral line shapes,^16^ whereas the observed PCr linewidth for AW SLAM and AW SLIM is not significantly different to AW FT‐MRS. The origin of the SNR increase seen here is different to the previous body of work on SLAM,^20^ which used a reduced set of high SNR central k‐space phase encodes to achieve a per unit volume increase in SNR over a higher resolution uniform set of k‐space phase encodes. However, the increase in sensitive volume while maintaining the PCr/ATP ratio is only possible because of the compartment‐based reconstruction producing a cardiac compartment SRF, which closely matches the shape of the heart, unlike the summation of AW FT‐MRS voxels (see the supporting information).

These results, particularly when combined with the calculated AW SRFs, suggest that the AW SLAM and AW SLIM methods afford improved performance compared with AW FT‐MRS for cardiac ^31^P MRS at 7 T where B_0_ and B_1_ inhomogeneity are of greater concern than at 3 T.

### 5.2 Truncated k‐space acquisitions

4 × 4 × 4 SLAM and 4 × 4 × 4 SLIM SNRs were significantly increased over the AW FT‐MRS method, although neither 4 × 4 × 4 SLAM nor 4 × 4 × 4 SLIM showed improved reproducibility (CoV and CoR) compared to AW FT‐MRS. As expected, optimizing the 4 × 4 × 4 phase encode scheme on a per‐volunteer basis with the fSLAM optimization algorithm improved both CoV and CoR compared with the 4 × 4 × 4 SLAM and 4 × 4 × 4 SLIM methods; however, the CoV and CoR for fSLAM were still higher than for the AW SLAM method. Overall, we were not able to demonstrate a significant benefit to using 4 × 4 × 4 SLAM, 4 × 4 × 4 SLIM, or fSLAM methods rather than the AW SLAM method, because the improved SNR did not translate to improved reproducibility.

### 5.3 SLAM and SLIM reconstruction algorithms

Both SLAM and SLIM reconstructions significantly increased PCr SNR compared to AW FT‐MRS, but without significantly affecting either the PCr/ATP ratio or PCr resonance linewidth. This is probably because of an increase in the coil sensitivity‐weighted tissue volume to incorporate a greater proportion of the heart, without including the chest wall, potentially increasing sensitivity to motion. The calculated SRFs suggest that cardiac/chest wall movement through breathing may be an important factor to consider for both SLAM and SLIM algorithm‐based methods. Because the SRF is much larger than for a conventional Fourier‐based reconstruction, it is more likely that the chest wall will stray into the cardiac SRF during breathing, potentially leading to contamination of the cardiac spectra. While this may present an issue, AW SLAM and AW SLIM each had a lower CoV and CoR than the AW FT‐MRS method, and cardiac/respiratory gating, in addition to scanning in the prone position, may ameliorate this concern further.

## 6 CONCLUSION

In this study, we explored the feasibility of using SLAM and SLIM reconstruction techniques for 3D ^31^P cardiac spectroscopy at 7 T. Our main finding is that it is possible to achieve an increase in SNR over the conventional AW FT‐MRS method, without significantly affecting the measured PCr/ATP ratio, while improving both the coefficients of variation and reproducibility (using AW SLIM and AW SLAM). We also note that the AW acquisition scheme is already commonly used, greatly simplifying and de‐risking clinical implementation of these reconstruction algorithms.

## Supporting information


**Figure S1.** The summed point spread function (PSF) of voxels corresponding to the chest wall are shown on the left and right (note difference to SRFs shown in main body text). The receive sensitivity map is shown in the centre. The cardiac voxels are highlighted with the white bounding line and the midseptal voxel is additionally highlighted with a white bounding box in the centre of the heart. Contamination of the voxels forward of the midseptal voxel by the chest wall signal is evident, as is the increased coil sensitivity in this region.
