# Supplementary material for: Compartment‐based reconstruction of 3D acquisition‐weighted 31P cardiac magnetic resonance spectroscopic imaging at 7 T: A reproducibility study
Source: NMR Biomed. 2023 May 4;36(9):e4950. doi: 10.1002/nbm.4950 (PMC10658645; doi:10.1002/nbm.4950)
Supplement: Supplementary file 1 — Figure S1. The summed point spread function (PSF) of voxels corresponding to the chest wall are shown on the left and right (note difference to SRFs shown in main body text). The receive sensitivity map is shown in the centre. The cardiac voxels are highlighted with the white bounding line and the midseptal voxel is additionally highlighted with a white bounding box in the centre of the heart. Contamination of the voxels forward of the midseptal voxel by the chest wall signal is evident, as is the increased coil sensitivity in this region. [file NBM-36-e4950-s001.docx]

Sum of AW FT-MRS voxels

Summation of spectra, reconstructed with the FT-MRS method, corresponding to voxels located within the heart, may increase the SNR of the reconstructed cardiac spectrum, by expanding the size of the sensitive volume, however taking voxels close to the chest wall may lead to increased chest wall PCr contamination.

To test the effect of summing cardiac voxels the following experiment was performed. The AW FT-MRS method was modified so instead of taking just the midseptal voxel, all voxels corresponding to the heart compartment were individually phased and summed together. The calculated statistics were as follows: PCr/ATP = 2.22 ± 0.49*, SNR = 34.32 ± 16.61*, PCr line-width = 53.16 ± 26.13 Hz*, CRLB = 4.42 ± 1.86, CoR = 0.66, CoV = 0.22. (* indicates significantly different to the AW FT-MRS method – paired Wilcoxon sign-rank test p = 5%)

**Figure S1:** The summed point spread function (PSF) of voxels corresponding to the chest wall are shown on the left and right (note difference to SRFs shown in main body text). The receive sensitivity map is shown in the centre. The cardiac voxels are highlighted with the white bounding line and the midseptal voxel is additionally highlighted with a white bounding box in the centre of the heart. Contamination of the voxels forward of the midseptal voxel by the chest wall signal is evident, as is the increased coil sensitivity in this region.

The high SNR and low CoR and CoV, relative to AW FT-MRS can be explained as follows. When the spectra are summed, the spectra originating from voxels close to the chest wall, which have high SNR due to the coil B_1_ profile (Figure S1), dominate the resultant spectra, giving it high SNR and consequently low CoR and CoV. However, the spectra from voxels close to the chest wall are also contaminated by the strong chest wall PCr signal, meaning that the summed spectra has an artificially inflated PCr resonance, leading to a PCr/ATP which is significantly higher than AW FT-MRS. This makes summation of all (or a subset of high SNR) cardiac voxels inadvisable, as the resultant spectra will not reflect the true underlying cardiac PCr/ATP ratio.

Although the worsening of CoR seen by Ellis et al.[*NMR in Biomed*. 2019; 32:e4095] when summing up to four voxels, is contrary to this result, we hypothesize that this due to the use of saturation bands in their study, which give a relatively sharp cut-off to chest wall PCr contamination, making the exact voxel position (which would vary between scan 1 and scan 2 of the test re-test procedure) critical.

Full segmentation of the heart


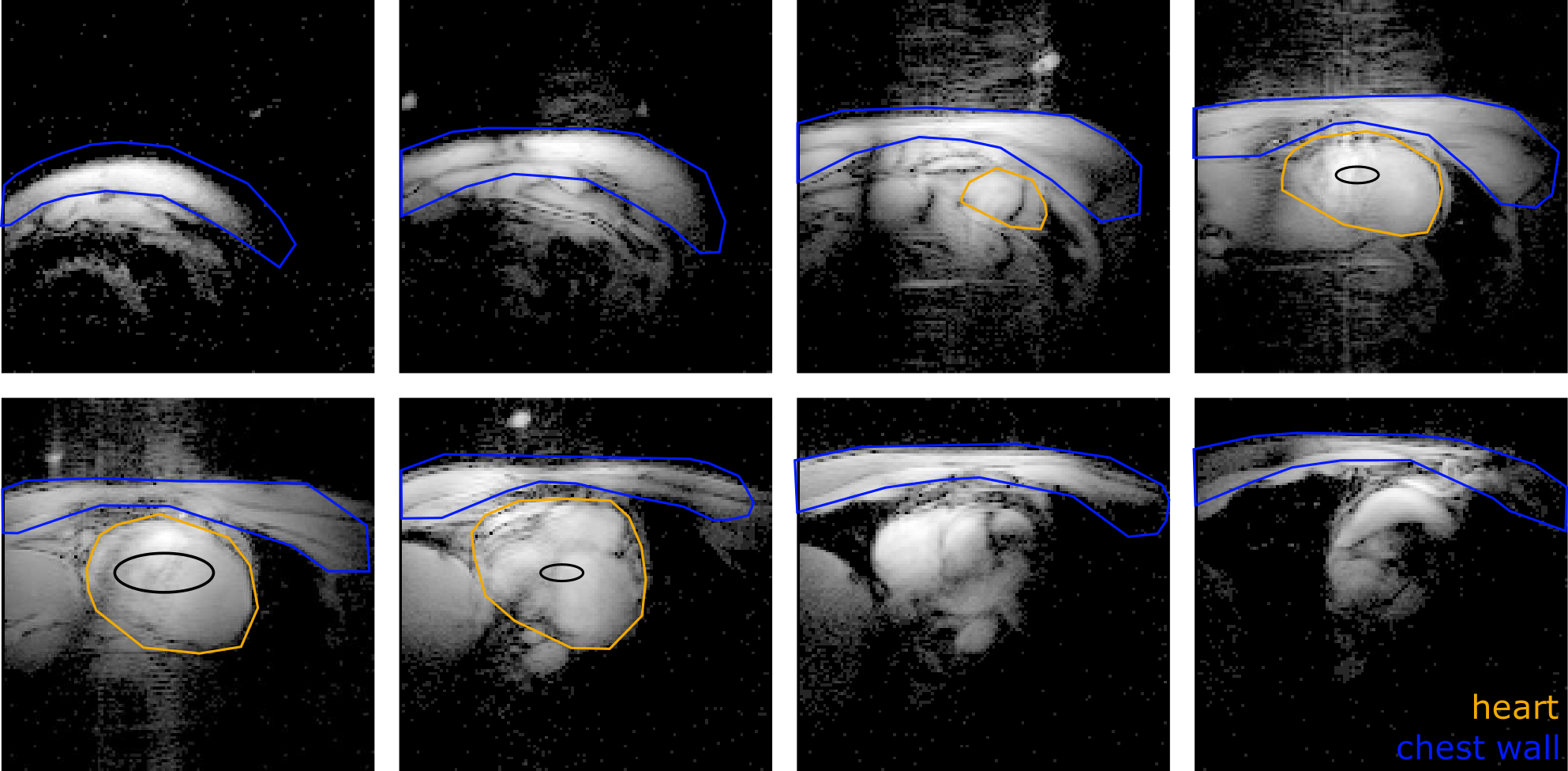


This figure shows a full segmentation of the heart for one volunteer, the heart compartment is shown in orange and the chest wall in blue, with the 64% threshold of the AW FT-MRS PSF shown in black. Note that the segmentation was performed scanner side during the acquisition of the AW dataset, to enable calculation of the fSLAM phase encode values.
